# United States Consul Worman and His Work

**Published:** 1901-12

**Authors:** 


### UNITED STATES CONSUL WORMAN AND HIS WORK.

The following abstract of a letter received from United States Consul James H. Worman will be of interest to all our readers. He says, "I am delighted to report to you this morning (October 30) that the Bavarian ministry has favorably answered our petition to recognize the degrees of reputable American dental colleges. I hope to carry this work successfully with all the states of Germany. This news will be of special interest to you, as it confirms my stand taken before the National Association of Dental Faculties in August, that it is our task to do away with frauds here in order to inspire with confidence in our educational institutions the governments abroad.

(Signed) "James H. Worman."

This acceptance of the diplomas of reputable colleges of the United States by the Bavarian government will unquestionably have a marked influence upon the other states composing the German Empire, but whether they will go as far as Bavaria has gone is doubtful. This state has always been more liberal than some of the others in its recognition of outside educational work.

It is, however, none the less gratifying that this recognition has been accorded, and may it be hoped that this will prove an entering influence that will eventually establish the educational systems of all countries upon a reciprocal basis. The world is fast coming to a period when the narrow distinctions of national boundary lines should be obliterated as far as professional educational attainments are concerned. The diploma from a recognized institution should be a passport everywhere to admit the bearer to active work among all civilized peoples. While the states of this government persist in erecting barriers to practice against the residents of other states, it does not become us to criticise foreign governments for doing the same thing, and thereby protect their people from outside encroachments. When these facts are considered, the liberality of the Bavarian government can be more fully appreciated.

This action must be ascribed to the earnest work of Consul Worman. Those who are familiar with his very exhausting labors in this direction need not to be told that his influence in Bavaria has had largely to do with this decision. His valuable work in exposing the fraudulent colleges in this country has made his name conspicuous both at home and abroad, and it has had a marked influence in governmental circles. The government at Washington has been kept fully informed of his work from the beginning. His writings in this direction have been voluminous, the report of his past summer's work alone covering over two hundred pages.

When it is considered that Consul Worman has neither personal nor professional interest in this labor, that he is spending time, money, and strength simply that American degrees may be purified from all taint of fraud, and be thereby honored for all that they are really worth the world over, it becomes of exceptional interest; and when it is considered that his work has been mainly to cover the dental degree with the mantle of his powerful influence, the members of the dental profession should not hesitate to express in unmistakable terms the appreciation they entertain for his accomplished results, and the unselfish motives that have actuated it from the beginning.

It has been mainly through the evidence furnished by him that the recent prosecutions in Illinois have been so successful, and this will be further continued until the gang of diploma conspirators have been completely routed.

The dentists of Chicago and Illinois seem to have at last awakened to the fact that they have something to do in this direction: it is not much to their credit that this inspiring force had to come through a United States Consul. It seems to an outsider that a little home effort might have accomplished earlier results.

This journal has been sharply criticised in certain quarters for its supposed opposition to this work of the Association of Faculties. This is a misapprehension of the motives of the writer. The earlier work of the Foreign Relations Committee was warmly endorsed, inasmuch as its chairman energetically opened the way to the suppression of the fraudulent diploma business in Chicago; but this having been accomplished, the work should have been left in the hands of the police authorities of that city, as it was not the duty of an educational body to continue that labor. That opinion is still held, and in the writer's judgment the Faculties Association lowers its standard by meddling with the duty of the constituted authorities. While this is true, it does not follow that this body may not aid financially, or in any other way consistent with its position as a leader in educational thought and practice. It was, therefore, proper to raise a certain fund to assist in uprooting this evil, but beyond this it ought not to go. There is a wide difference between this position and that of Consul Worman, or that of individuals, and his work, and that of those who are aiding the authorities of the State of Illinois in bringing these men to the halls of justice to there meet their just deserts, will continue to receive the cordial endorsement of this journal. The Association of Faculties was organized to raise the standard of professional training in all the dental colleges of this country. That it has succeeded in this to a notable degree is known to all; but it must confine its labors within certain well-defined limitations, or it will deteriorate, and its moral influence, heretofore so powerful, will be measurably lost.

The members of the Faculties Association will naturally feel a deep interest in the way the funds ordered through assessment will be appropriated. They have confidence in the men having this in charge, but some, at least, will not be satisfied if the Foreign Relations Committee undertake to manage the prosecutions as a representative body of the Faculties Association. It would seem eminently proper that this committee should aid Consul Worman to the fullest extent, for it is an injustice to him that he should be forced to spend his private funds in the interest of dentistry of this country. He has already sacrificed a large sum in this direction, and that without hope of reward save the consciousness of duty performed. If he succeeds in destroying the last remnants of this nefarious traffic in diplomas, he will have accomplished more than the police authorities of States and cities have been able to do in the past half century. While he is still energetically at work, let the dental profession of this country give him undivided support and hold him personally in the highest honor.

